# Stabilization of modulation instability by control field in semiconductor quantum wells

**DOI:** 10.1038/s41598-023-34867-5

**Published:** 2023-05-11

**Authors:** Monika Nath, Rohit Mukherjee, Nitu Borgohain

**Affiliations:** 1 Department of Physics, University of Science & Technology Meghalaya, Ri-Bhoi, India 793101; 2 Department of Physics, Sarala Birla University, Jharkhand, India 835103

**Keywords:** Optics and photonics, Optical physics

## Abstract

This article explores the modulation instability of a continuous or quasi-continuous weak probe pulse in a three-level asymmetric double quantum wells under an electromagnetically induced transparency regime, controlled by a strong laser beam. The dynamics of modulation instability reveals that the instability gain as well as its bandwidth is greatly influenced by control field Rabi frequency. The probe pulse is found to be almost stable against modulation instability for higher values of control field Rabi frequency. The results of this investigation may potentially apply for oscillation free generation of supercontinuum in quantum well nanostructures.

## Introduction

Modulation instability (MI) is a fundamental phenomenon associated with an exponential growth of weak perturbed waves, during their propagation through nonlinear dispersive media^1–6^. It occurs due to the interaction of nonlinear and dispersive effects which lead to the disintegration of waves into a train of shape preserving ultrashort pulses, called solitons^7–9^. In frequency domain, it causes transfer of energy from a strong single spectral component onto sidebands, while in space, it transforms weakly modulated plane waves into spatial periodic waves^10–12^. Studies and experiments on MI have started almost simultaneously across the world around 1965’s. The earlier approach to solving MI follows the classical Lighthill criterion, which is considered when nonlinearity and dispersion make opposite contribution to wave frequency^13,14^. Numerous investigations on wave instability in optical fibers reveal wave mixing instabilities that are not addressed by the Lighthill criterion, were solved using generalized nonlinear Schrödinger equation (NLSE)^15^. Unstable propagation of continuous (CW) or quasi-continuous waves (QCW) in nonlinear media possessing Kerr nonlinearity and higher-order dispersion is found to be responsible for generation of spectral sidebands^16–18^. These sidebands further supports in the generation of supercontinuum (SC) in few selected optical media^19–22^. MI has received special attention in optical fibers, since they possess high nonlinearity and tailorable dispersion profile. The first experimental observation of MI effect was reported by Tai et al.^23^ in 1986. Extension of MI to the normal dispersion regime was predicted in 1970^24^. Since, then numerous researches were exerted towards improvement of MI spectra. A decade back, Erkintalo et al.^25^ have reported a theoretical and experimental study on higher order MI in optical fibers. MI in dispersion oscillating fibers has reported by Mussot et al.^20^. Recently, Kraych have reported a unique behavior in the evolution of a modulationally unstable plane wave driven by a small noise in fiber optics^26^. In a recent study, Liu et al. have reported to obtain asymmetric spectra of MI by applying asymmetric physical effect called self-steepening^27^.

Since the advent of working example of photonic crystal fibers (PCFs)^28^ in 1996, MI study has geared up in these media extensively, taken into account the flexibility to adjust the dispersion profile. Though initially, the MI was studied in anomalous dispersion regime, but in 2003 Harvey et al.^29^ have demonstrated the role of MI in the normal dispersion regime also. Chun et al. in 2003 further reported that MI can occur both in normal and anomalous dispersion regime^30^. With the support of MI assisted four-wave mixing (FWM), broad and tunable multi-wavelength fiber laser was proposed by Liu^31^ in 2010. Investigation of MI in PCFs pick up pace due to the generation of broad range of new frequencies leading to the generation of SC^32–35^.

Though the investigation of MI is mostly centered in optical fibers and PCFs, but the identification of enhanced nonlinearity and dispersion in solid state media, especially, in semiconductor quantum wells (SQWs) attains immense attraction, very recently^36,37^. This keen interest in SQWs is due to their inherent properties over other optical media, such as large optical dipole moments, which leads to giant nonlinear coefficients and widely adjustable factors that offer large suppleness in device design. On top of that, many nonlinear optical phenomena investigated by quantum interference and coherence effect, such as, electromagnetically induced transparency (EIT), lasing without inversion, ultraslow light, Kerr nonlinearity, optical soliton etc., have been efficiently obtains in SQWs^36,38–40^. Recently, Mukherjee et al. have investigated the effects of quintic nonlinearity on MI in multiple coupled quantum wells^41^, they in a landmark work^42^ also have studied the dynamics of MI under the effect of relative phase of the applied optical fields in EIT regime. Borgohain et al.^37^ have examined how the detuning of the control field effects on the growth of MI in three-level symmetric quantum wells, by incorporating higher-order nonlinearities and dispersions. Since till date, it is well established that the control field parameters, operated under EIT are the key to manipulate or enhance the nonlinearity of SQW systems which leads to numerous applications in nonlinear optics and photonics^36,37^. In view of this, it is worth investigating the effect of the control field intensity on the growth or stabilization of MI in SQWs, which is not yet addressed by any researchers. Therefore, the main thrust of this paper is to study the MI of a continuous or quasi-continuous weak probe field under the effect of control field intensities in an asymmetric double quantum wells.

In this paper, we present the theoretical model and the master equations of asymmetric double quantum wells interacting with a continuous or quasi-continuous probe and a strong control laser field. The expression of the dispersions, nonlinear Schrödinger equation, and the analytical expression of modulation instability gain are also derived. In addition, the properties of linear and Kerr nonlinearity, and the investigation of MI under the variation of power of the optical probe and control field Rabi frequencies are investigated.

## Mathematical model and governing equations

**Figure 1:**
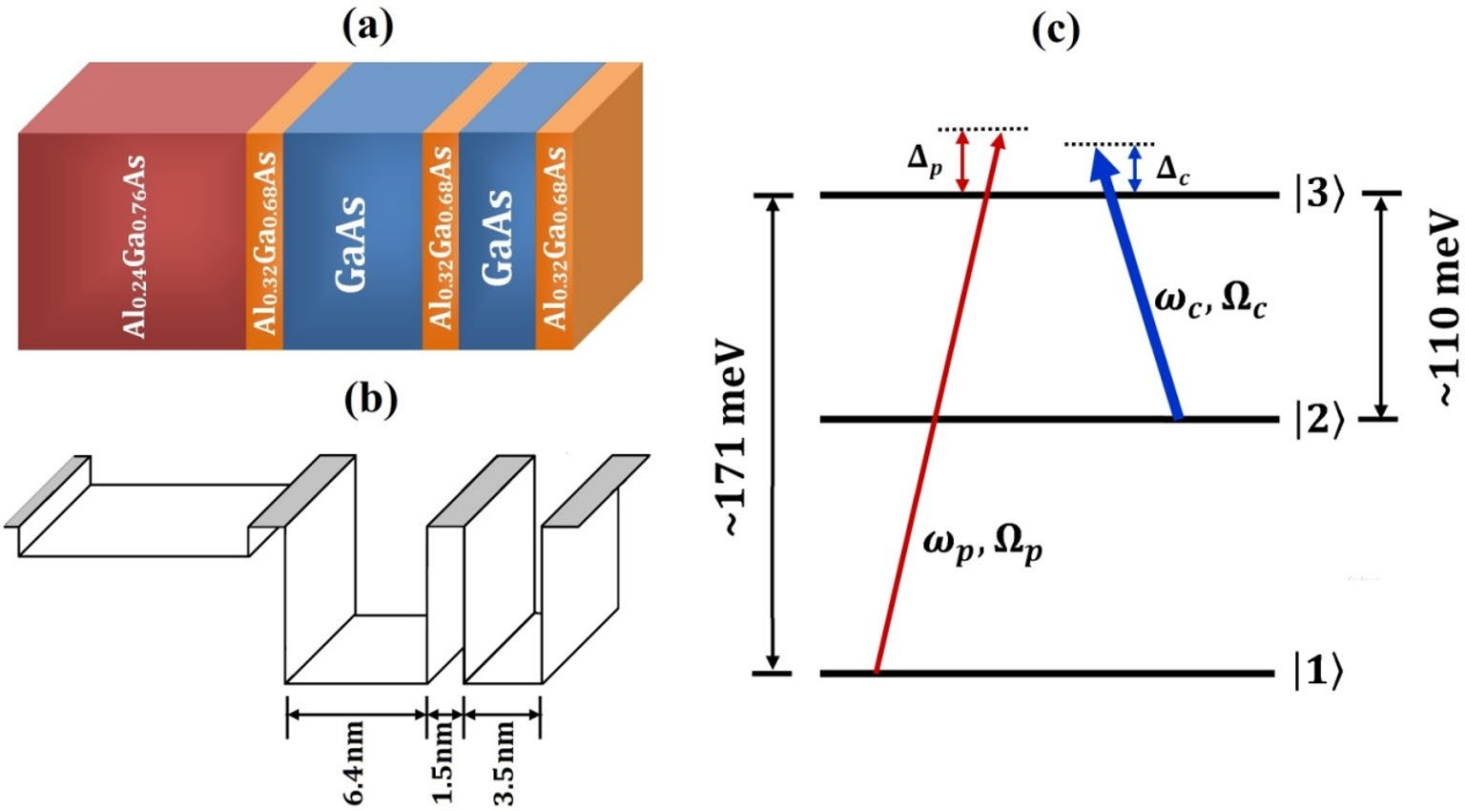
(**a**) Schematic diagram of a single period of ADQWs, (**b**) quantum well profile, (**c**) Energy level diagram for transition.

A three-level asymmetric double quantum wells (ADQWs)^43^ is considered, which undergoes with a Λ-type configuration as depicted in Fig. 1. This chosen structure consists of 1.5 nm ${\text{Al}}_{0.32}{\text{Ga}}_{0.68}{\text{As}}$ tunnel barrier separating two GaAs well layers of thickness 6.4 nm and 3.5 nm, respectively. There is a 1.0 nm ${\text{Al}}_{0.32}{\text{Ga}}_{0.68}{\text{As}}$ barrier on the left-hand side of the wide well followed by a thick layer of ${\text{Al}}_{0.24}{\text{Ga}}_{0.76}{\text{As}}$ on the left, and finally ${\text{Al}}_{0.32}{\text{Ga}}_{0.68}{\text{As}}$ potential barrier on the left of narrow well. Here, left side of the ADQWs is chosen as buffer layer made of ${\text{Al}}_{024}{\text{Ga}}_{0.76}{\text{As}}$ which can be considered as a continuum region of the double quantum wells. The advantage of the continuum layer is to prevent any resonant tunneling between the adjacent quantum wells^44^. To investigate the optical response of the ADQWs, a weak probe field of amplitude ${E}_{p}$ and central frequency ${\omega }_{p}$ is applied to the transition $\left|1\rangle \right.\to \left|3\rangle \right.$, with intersubband^45^ transition energy ~ 171 meV. Simultaneously, a stronger control field with amplitude ${E}_{c}$ and central frequency ${\omega }_{c}$ is applied to the transition $\left|2\rangle \right.\to \left|3\rangle \right.$, with intersubband transition energy ~ 110 meV. In this investigation, for optical transition, the values of energy levels can be obtained by solving the effective mass Schrödinger equation^43^. However, for obtaining intersubband transition energies few other suitable approximations may also be used, such as energy band nonparabolicity and effective mass approximation^46^. For present study, the intersubband transition energies are picked up from available literature^43^. The semi-classical Hamiltonian of the system can be written under the rotating wave approximation^37^ as

1 $$\widehat{H}=\sum_{i=1}^{3}\hbar{{\omega}}_{i}|i\rangle \langle i|-\hbar\left\{{\Omega }_{p}{e}^{i\left({k}_{p}z-{{\omega}}_{p}t\right)}\left|3\rangle \langle 1\right|+{\Omega }_{c}{e}^{i\left({k}_{c}z-{{\omega}}_{c}t\right)}\left|3\rangle \langle 2\right|+h.c.\right\},$$

here, the first two terms signify the free Hamiltonian and light-matter interaction part, h.c. stands for Hermitian conjugate. The Rabi-frequencies of the weak probe and strong control are expressed as ${\Omega }_{p}=\frac{{\widehat{\mu }}_{31}{\widehat{e}}_{p}{E}_{p}}{\hbar}$ and ${\Omega }_{c}=\frac{{\widehat{\mu }}_{32}{\widehat{e}}_{c}{E}_{c}}{\hbar}$, respectively.

In order to study light-matter interaction problem in coupled quantum well systems, we follow the density matrix method^47^ and obtain the set of Maxwell’s Bloch equations:

2 $$\frac{\partial }{\partial t}{\widetilde{\rho }}_{11}=-i{\Omega }_{p}{\widetilde{\rho }}_{13}+i{\Omega }_{p}^{*}{\widetilde{\rho }}_{31},$$

3 $$\frac{\partial }{\partial t}{\widetilde{\rho }}_{22}=-i{\Omega }_{c}{\widetilde{\rho }}_{23}+i{\Omega }_{c}^{*}{\widetilde{\rho }}_{32}-{\gamma }_{2}{\widetilde{\rho }}_{22},$$

4 $$\frac{\partial }{\partial t}{\widetilde{\rho }}_{33}=i{\Omega }_{p}{\widetilde{\rho }}_{13}-i{\Omega }_{p}^{*}{\widetilde{\rho }}_{31}+i{\Omega }_{c}{\widetilde{\rho }}_{23}-i{\Omega }_{c}^{*}{\widetilde{\rho }}_{32}-{\gamma }_{3}{\widetilde{\rho }}_{33},$$

5 $$\frac{\partial }{\partial t}{\widetilde{\rho }}_{21}=i\left({\Delta }_{p}-{\Delta }_{c}\right){\widetilde{\rho }}_{21}-i{\Omega }_{p}{\widetilde{\rho }}_{23}+i{\Omega }_{c}^{*}{\widetilde{\rho }}_{31}-\frac{1}{2}{\gamma }_{12}{\widetilde{\rho }}_{21},$$

6 $$\frac{\partial }{\partial t}{\widetilde{\rho }}_{31}=i{\Omega }_{p}{\widetilde{\rho }}_{11}-i{\Omega }_{p}^{*}{\widetilde{\rho }}_{33}+i{\Omega }_{\mathrm{c}}{\widetilde{\uprho }}_{21}-\frac{1}{2}{\gamma }_{13}{\widetilde{\rho }}_{31}+i{\Delta }_{p}{\widetilde{\rho }}_{31},$$

7 $$\frac{\partial }{\partial t}{\widetilde{\rho }}_{32}=i{\Omega }_{p}{\widetilde{\rho }}_{12}+i{\Omega }_{c}{\widetilde{\rho }}_{22}-i{\Omega }_{c}{\widetilde{\rho }}_{33}-\frac{1}{2}{\gamma }_{23}{\widetilde{\rho }}_{32}+i{\Delta }_{c}{\widetilde{\rho }}_{32}.$$

Here, ${\widetilde{\rho }}_{11}={\rho }_{11}, {\widetilde{\rho }}_{22}={\rho }_{22}, {\widetilde{\rho }}_{33}={\rho }_{33}, {\widetilde{\rho }}_{21}={\rho }_{21}{e}^{i\left({{\omega}}_{p}-{{\omega}}_{c}\right)t}, {\widetilde{\rho }}_{31}={\rho }_{31}{e}^{{{\omega}}_{p}t},$ and ${\widetilde{\rho }}_{32} ={\rho }_{32}{e}^{{i{\omega}}_{c}t}$ are the rapid oscillating terms. Owing to the low temperature longitudinal optical phonon emission, scattering of electron by electron, and scattering of electron by phonon at the interfaces, the transitions in ADQWs suffers the population decay rates, given by parameter ${\gamma }_{ij} (={\gamma }_{i}+{\gamma }_{ij}^{dph})$. Under steady state condition, the solution of the density matrix elements leads to the expressions of susceptibility of the probe pulse. Within adiabatic framework and weak probe field approximation^47–49^, $\left|{\Omega }_{p}\right|\ll |{\Omega }_{c}|$, for which the depletion of the ground state is minimum at $t>0$ i.e., ${\widetilde{\rho }}_{11}\approx 1, {\widetilde{\rho }}_{ii}\approx 0 \left(i=2, 3\right)$. Therefore, a perturbative expansion ${\widetilde{\rho }}_{ij}=\sum_{k}{\widetilde{\rho }}_{ij}^{\left(k\right)}$, is followed, where, ${\widetilde{\rho }}_{ij}^{\left(k\right)}$ is the k-th order perturbation of ${\widetilde{\rho }}_{ij}$. Hence, under the adiabatic approximation, it is simple to determine ${\widetilde{\rho }}_{ij}^{\left(0\right)}={\delta }_{ij}, {\widetilde{\rho }}_{i1}\cong {\widetilde{\rho }}_{i1}^{\left(1\right)}{\widetilde{\rho }}_{11}^{\left(0\right)}$, and ${\widetilde{\rho }}_{ii }^{\left(0\right)}\cong 0 (i=2, 3)$ i.e., ${\widetilde{\rho }}_{11}\simeq 1 ,{\widetilde{\rho }}_{ii}\simeq 0$. Taking Fourier transformation of Eqs. (5) and (6) according to following rule ${\widetilde{\rho }}_{i1}^{\left(1\right)}=\frac{1}{\sqrt{2\pi }}{\int }_{-\infty }^{+\infty }{\beta }_{i1}^{\left(1\right)}{e}^{-i{\omega}t}d{\omega},$ and ${\Omega }_{p}=\frac{1}{\sqrt{2\pi }}{\int }_{-\infty }^{+\infty }{\Lambda }_{p}{e}^{-i{\omega}t}d{\omega} ,$ we obtain

8 $${{\beta }_{21}^{(1)} =\frac{{\Lambda }_{p}{\Omega }_{c}^{*}}{D({\omega})},\text{ and} \beta }_{31}^{(1)}=-\frac{{\Lambda }_{p}{D}_{p}\left({\omega}\right)}{D\left({\omega}\right)},$$

here, ${\beta }_{i1}^{\left(1\right)}$ and ${\Lambda }_{p}$ designates the Fourier transform of ${\widetilde{\rho }}_{i1}^{\left(1\right)}$ and ${\Omega }_{p};$ where ${\omega}$ signifies the Fourier transform parameter with ${D}_{p}\left({\omega}\right)=\left\{{\omega}+\left({\Delta }_{p}-{\Delta }_{c}\right)+i\frac{{\gamma }_{21}}{2}\right\},$ and $D\left(\omega {\omega}\right)=\left\{{\omega}+\left({\Delta }_{p}-{\Delta }_{c}\right)+i\frac{{\gamma }_{21}}{2}\right\}\{{\omega}+({\Delta }_{\mathrm{p}}+i\frac{{\upgamma }_{31}}{2})-{\left|{\Omega }_{\mathrm{c}}\right|}^{2}\}$. By using inverse Fourier transformation, we arrive at

9 $${\widetilde{{\widetilde{\rho }}_{21}^{\left(1\right)}=\frac{{\Omega }_{\mathrm{p}}{\Omega }_{\mathrm{c}}^{*}}{D\left(0\right)}, \, \text{ and}  \, \rho }}_{31}^{\left(1\right)}=-\frac{{\Omega }_{\mathrm{p}}{\mathrm{D}}_{\mathrm{p}}\left(0\right)}{D\left(0\right)}.$$

The induced polarization $P$ around frequency ${\omega }_{p}$ controls the response of the probe field in ADQWs, can be expressed as $P={\varepsilon }_{o}{\chi }_{p}{E}_{p},$ where ${\varepsilon }_{o}$ is the permittivity of free space and ${\chi }_{p}$ is the optical susceptibility of probe field at frequency ${\omega }_{p}$. Hence, the susceptibility ${\chi }_{p}$ can be expressed as:

10 $${\chi }_{p}=\frac{N{\left|{\widehat{\mu }}_{31}\right|}^{2}}{{\epsilon }_{0}{\Omega }_{p}\hbar}\cdot {\widetilde{\rho }}_{31}=\frac{N{\left|{\widehat{\mu }}_{31}\right|}^{2}}{{\epsilon }_{0}{\Omega }_{p}\hbar}\left[{\widetilde{\rho }}_{31}^{\left(1\right)}-{\widetilde{\rho }}_{31}^{\left(1\right)}\left({\left|{\widetilde{\rho }}_{21}^{\left(1\right)}\right|}^{2}+{\left|{\widetilde{\rho }}_{31}^{\left(1\right)}\right|}^{2}\right)\right].$$

Further, the susceptibility ${\chi }_{p}$ can be decomposed into linear and nonlinear parts as

11 $${\chi }_{p}={\chi }^{(1)}+{\chi }^{(3)}{\left|{E}_{p}\right|}^{2}+\dots $$

Substituting the values of ${\widetilde{\rho }}_{21}^{\left(1\right)}$ and ${\widetilde{\rho }}_{31}^{\left(1\right)}$ in Eq. (10), and we obtain

12 $${\chi }^{\left(1\right)}=-\frac{N{\left|{\widehat{\mu }}_{31}\right|}^{2}}{\hbar{\epsilon }_{0}}\cdot \frac{{D}_{p}(0)}{D(0)}$$

13 $${\chi }^{(3)}=\frac{N{\left|{\widehat{\mu }}_{31}\right|}^{4}}{{\hbar}^{3}{\epsilon }_{0}}\cdot \frac{{(|\Omega }_{c}^{*}{\left. \right|}^{2}+{\left|{D}_{p}\left(0\right)\right|}^{2})}{{\left|D\left(0\right)\right|}^{2}}\cdot \frac{{D}_{p}(0)}{D(0)}$$

Equations (12) and (13) are the simplified expressions for linear and third-order (or Kerr) susceptibilities. The linear susceptibility of the system is mainly responsible for attenuation and modulation of refractive index, whereas, the nonlinear susceptibility primarily drives several phenomena such as self-phase modulation (SPM)^41,50^, cross-phase modulation (XPM)^48^, FWM^49^ etc.

## Linear dispersion and nonlinear effect

The evolution of the weak probe field during propagation through the ADQWs is governed by Maxwell’s wave equation as

14 $${\nabla }^{2}\overrightarrow{E}-\frac{1}{{C}^{2}}\frac{{\partial }^{2}\overrightarrow{E}}{{\partial t}^{2}}=\frac{1}{{\varepsilon }_{0}{c}^{2}}\frac{{\partial }^{2}\overrightarrow{P}}{{\partial t}^{2}}$$

Considering the probe field to be homogeneous along transverse direction and considering slowly varying envelope approximation, the Eq. (14) reduces to

15 $$\frac{\partial {\Omega }_{p}}{\partial z}+\frac{1}{C}\frac{\partial {\Omega }_{p}}{\partial t}=\frac{i{{\omega}}_{p}{|{\widehat{\mu }}_{31}|}^{2}N {\widetilde{\rho}}_{31}}{2{\epsilon }_{0}c \hbar}=\mathrm{i\alpha }{\widetilde{\rho}}_{31}$$

where, $\mathrm{\alpha }=\frac{\mathrm{N}{|{\widehat{\mu }}_{31}|}^{2}{{\omega}}_{\mathrm{P}}}{2{\hbar}{\upepsilon }_{0}\mathrm{c}}$ with reduced Plank’s constant $\hslash$ and $N$ is the carrier density of the ADQWs.

To introduce the dispersion effect, we performed the Fourier transformation of Eq. (15) as

16 $$\frac{\partial {\Lambda }_{p}}{\partial z}-i\beta ({\omega}){\Lambda }_{p}=0$$

where, the propagation constant $\beta (\omega)$ can be written as $\beta ({\omega})=\frac{{\omega}}{c}-\alpha \frac{{D}_{p}\left({\omega}\right)}{D({\omega})}$, which can be expressed in Taylor series expansion as

17 $$\beta \left(\omega\right)=\beta \left(0\right)+{\beta }_{1}\left(0\right){\omega}+\frac{1}{2}{\beta }_{2}\left(0\right){{\omega}}^{2}+\frac{1}{6}{\beta }_{3}\left(0\right){{\omega}}^{3}+ ..\dots .$$

Here,

18 $$\beta \left(0\right)=-\alpha \frac{{D}_{p}\left(0\right)}{D\left(0\right)}=-\alpha \frac{({\Delta }_{p}-{\Delta }_{c}+\frac{i{\gamma }_{21}}{2})}{\left({\Delta }_{p}+\frac{i{\gamma }_{31}}{2}\right)\left({\Delta }_{p}-{\Delta }_{c}+i\frac{{\gamma }_{21}}{2}\right)-{\left|{\Omega }_{c}\right|}^{2}}$$

19 $${\beta }_{1}\left(0\right)=\frac{1}{c}-\frac{\alpha \left({\omega}+{\Delta }_{p}-{\Delta }_{c}+\frac{i{\gamma }_{21}}{2}\right)}{\left({\omega}+{\Delta }_{p}-{\Delta }_{c}+\frac{i{\gamma }_{12}}{2}\right)\left({\omega}+{\Delta }_{p}+\frac{i{\gamma }_{13}}{2}\right)-{\left({\Omega }_{c}\right)}^{2}}$$

20 $$ \begin{aligned}{\beta }_{2}&=\frac{2\alpha (2{\omega}+2{\Delta }_{p}+\frac{i{\gamma }_{31}}{2}-{\Delta }_{c}+\frac{i{\gamma }_{21}}{2})}{{\left\{\left({\omega}+{\Delta }_{p}-{\Delta }_{c}+\frac{i{\gamma }_{21}}{2}\right)({\omega}+{\Delta }_{p}+\frac{i{\gamma }_{31}}{2}-{\left|{\Omega }_{c}\right|}^{2})\right\}}^{2}}\\ &\quad-\frac{2\alpha \left({\omega}+{\Delta }_{p}-2{\Delta }_{c}+\frac{i{\gamma }_{31}}{2}+\frac{i{\gamma }_{21}}{2}\right){\left(2{\omega}+2{\Delta }_{p}+\frac{i{\gamma }_{31}}{2}-{\Delta }_{c}\frac{i{\gamma }_{21}}{2}\right)}^{2}}{{\left\{\left({\omega}+{\Delta }_{p}-{\Delta }_{c}+\frac{i{\gamma }_{21}}{2}\right)\left({\omega}+{\Delta }_{p}+\frac{i{\gamma }_{31}}{2}-{\left|{\Omega }_{c}\right|}^{2}\right)\right\}}^{3}}\\ &\quad+ \frac{2\alpha (2{\omega}+2{\Delta }_{p}-{\Delta }_{c}+\frac{i{\gamma }_{31}}{2}+\frac{i{\gamma }_{21}}{2})}{{\left\{\left({\omega}+{\Delta }_{p}-{\Delta }_{c}+\frac{i{\gamma }_{21}}{2}\right)\left({\omega}+{\Delta }_{p}+\frac{i{\gamma }_{31}}{2}-{\left|{\Omega }_{c}\right|}^{2}\right)\right\}}^{2}}\end{aligned} $$

Physically, the imaginary part of $\upbeta \left(0\right)$ corresponds the absorption coefficient, and the real part to that of linear dispersion. ${\beta }_{1}(0)$ relates to group velocity of the probe field given as ${v}_{g}=Re(\frac{1}{{\beta }_{1}})$, while ${\beta }_{2}$ describes the probe group velocity dispersion (GVD), which causes the pulse to spread out in the temporal domain as it travels.

Now, keeping the terms of $\beta (\omega)$ up to second-order and considering the nonlinear part of ${\upbeta }_{31}^{\left(1\right)}$ we write

21 $$\left(\frac{\partial {\Lambda }_{\mathrm{p}}}{\partial \mathrm{z}}-\mathrm{i}\omega{\upbeta }_{1}{\Lambda }_{\mathrm{p}}-\frac{{\mathrm{i}}\omega^{2}}{2}{\upbeta }_{2}{\Lambda }_{\mathrm{p}}\right){\mathrm{e}}^{\mathrm{i\beta }(0)\mathrm{z}}=\left(NLT\right) {\upbeta }_{31}^{\left(1\right)}$$

Performing the inverse Fourier transformation of Eq. (21) as

22 $$\left(\frac{\partial {\Omega }_{\mathrm{p}}}{\partial \mathrm{z}}+{\upbeta }_{1}\frac{\partial {\Omega }_{\mathrm{p}}}{\partial \mathrm{t}}-\frac{1}{2}{\upbeta }_{2}\frac{{\partial }^{2}{\Omega }_{\mathrm{p}}}{\partial {\mathrm{t}}^{2}}\right)=\left(NLT\right) {\widetilde{\rho }}_{31}^{\left(1\right)}$$

here, the nonlinear parameter is defined as $NLT=-\mathrm{i\alpha }\left\{{\left|{\widetilde{\rho }}_{21}^{\left(1\right)}\right|}^{2}+{\left|{\widetilde{\rho }}_{31}^{\left(1\right)}\right|}^{2}\right\}.$

By virtue of Refs.^37,42^, and substituting the values of ${\widetilde{\rho }}_{21}^{\left(1\right)}$ and ${\widetilde{\rho }}_{31}^{\left(1\right)}$ in Eq. (22) gives

23 $$ i\frac{\partial {\Omega }_{\mathrm{p}}}{\partial \mathrm{z}}+\mathrm{i}{\upbeta }_{1}\frac{\partial {\Omega }_{\mathrm{p}}}{\partial \mathrm{t}}-\frac{1}{2}{\upbeta }_{2}\frac{{\partial }^{2}{\Omega }_{\mathrm{p}}}{\partial {\mathrm{t}}^{2}}+\mathrm{W}{\left|{\Omega }_{\mathrm{p}}\right|}^{2}{\Omega }_{\mathrm{P}}{\mathrm{e}}^{-\mathrm{az}}=0$$

where, $W=\alpha \{\frac{{\left|{\Omega }_{\mathrm{c}}^{*}\right|}^{2}+{\left|{\mathrm{D}}_{\mathrm{P}}\left(0\right)\right|}^{2}}{{\left|\mathrm{D}\left(0\right)\right|}^{2}}\}\cdot \frac{{D}_{p}(0)}{D(0)}$ arising due to Kerr nonlinearity. Under a retarded time frame given as $\xi =z$, $\eta =t-z{\beta }_{1},$ Eq. (23) reduces to

24 $$i\frac{\partial {\Omega }_{\mathrm{p}}}{\partial \mathrm{z}}-\frac{1}{2}{\upbeta }_{2}\frac{{\partial }^{2}{\Omega }_{\mathrm{p}}}{\partial {\mathrm{t}}^{2}}+\mathrm{W}|{{\Omega }_{\mathrm{P}}|}^{2}{\Omega }_{\mathrm{P}}=0$$

Equation (24) represents basic nonlinear Schrödinger equation of the CW or QCW probe field which predicts dynamics of MI in ADQWs.

### Modulation instability gain

To investigate the MI of the probe field, we follow a standard method^42,51,52^. To begin with, we consider a steady state solution for the NLSE (24), in the form of a CW or a QCW expressed as ${\Omega }_{p}\left(\xi ,t\right)=\left[\sqrt{{p}_{0}}+a(z,t)\right]{e}^{iW{p}_{0}z}$ which leads to

25 $$i\frac{\partial a}{\partial \xi }-\frac{1}{2}{\beta }_{2}\frac{{\partial }^{2}a}{\partial {T}^{2}}+W\sqrt{{p}_{0}}\left(a+{a}^{*}\right)\sqrt{{p}_{0}}=0$$

where, $\sqrt{{p}_{0}}$ represents the amplitude of the continuous wave with $a(z,t)$ being a small perturbation in the steady state.

Two side-band plane waves make up the general solution of Eq. (25) as

26 $$a\left(z,T\right)=U{e}^{i(K\xi -\Omega T)}+V{e}^{-i(K\xi -\Omega T)}$$

where, $\Omega$ and $K$ are the frequency shift and wave-vector, respectively. The amplitudes of the perturbed fields corresponding to Stokes and Anti-Stokes fields are $U$ and $V$. Substituting Eq. (26) into Eq. (25) we obtain a set homogeneous equations for $U$ and $V$ as

27 $$\left[\begin{array}{cc}-K+\frac{{\Omega }^{2}{\beta }_{2}}{2}+W{p}_{0}& W{p}_{0}\\ W{p}_{0}& K+\frac{{\Omega }^{2}{\beta }_{2}}{2}+W{p}_{0}\end{array}\right]\left[\begin{array}{c}U\\ V\end{array}\right]=0$$

The gain of the MI, $g(\Omega )$ is defined as $g\left(\Omega \right)=2Im(K)$, at any frequency $\Omega$. Therefore the MI gain spectrum turns out to be

28 $$g\left(\Omega \right)=\sqrt{4{\beta }_{2}{\Omega }^{2}W{p}_{0}-{\Omega }^{4}{\beta }_{2}^{2}} $$

The gain of the instability become maximum at frequency ${\Omega }_{max}=\sqrt{\frac{2{p}_{0}W}{{\beta }_{2}}},$ with ${g}_{max}=2W{p}_{0}.$ Now we are in a position to investigate the dynamics of MI of the probe field under the effect of the control field intensities.

## Results and discussions

### Linear and nonlinear properties of the ADQWs

**Figure 2:**
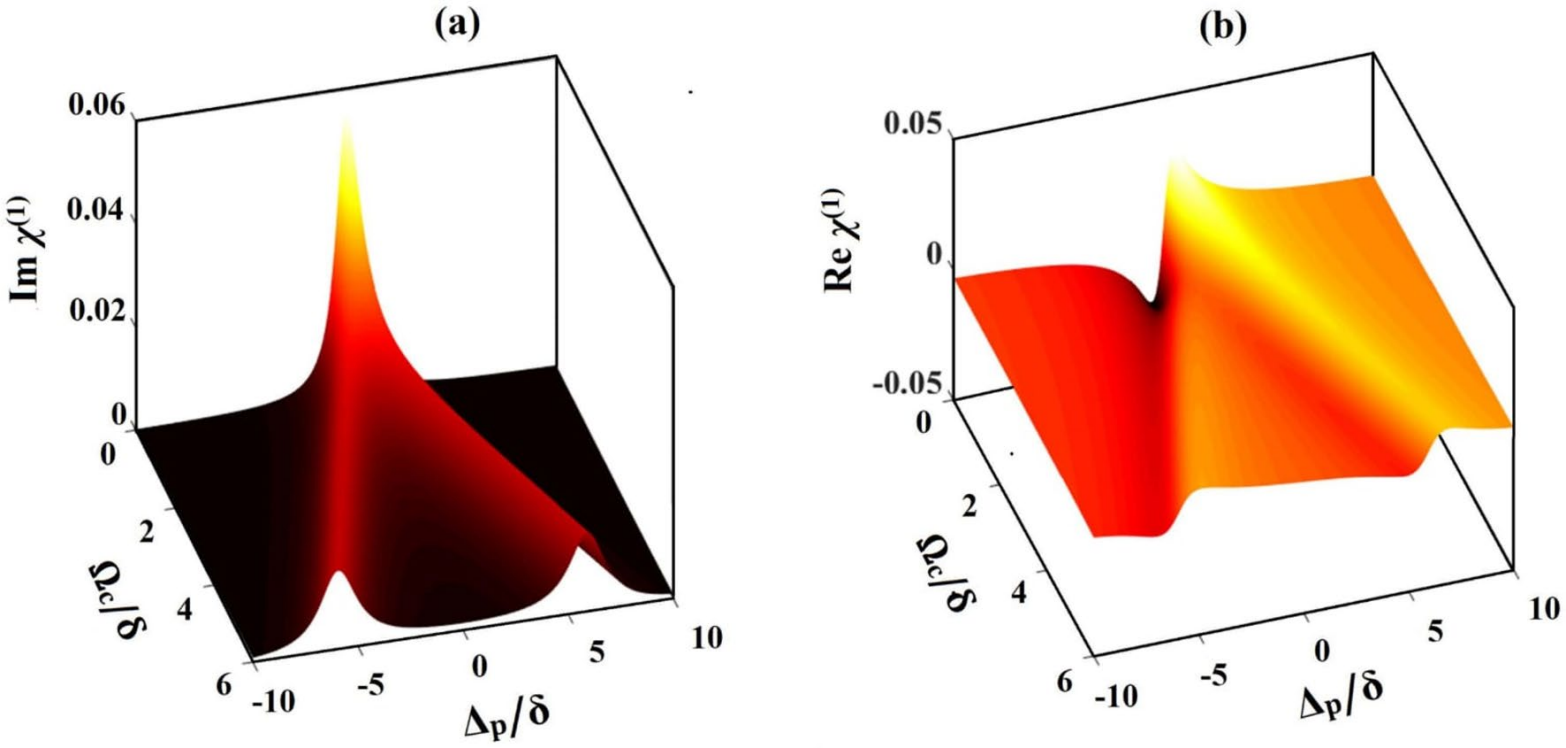
Three dimensional plot for (**a**) imaginary, and (**b**) real part of linear susceptibility against normalized Rabi-control frequency ${\Omega }_{c}/\delta$ and probe detuning ${\Delta }_{p}/\delta$.

The linear susceptibility of the system is related to absorption of probe field as well as modulation of the refractive index, whereas the nonlinear susceptibility gives rise to various nonlinear phenomena leading to new frequency generation in spectral domain. Therefore, it is crucial to first go through the linear and nonlinear susceptibilities of the ADQW systems. The system parameters used in the present investigation^43^ are: $N=3\times {10}^{23} \,  {\text{m}}^{-3}$,${\varepsilon }_{0}=8.85\times {10}^{-12} \, {\text{C}}^{2}{\text{N}}^{-1}{\text{m}}^{-2}$, ${\widehat{\mu }}_{31}=1.184\times {10}^{-29} \, {\text{Cm}}$, and the decay rates are ${\gamma }_{21}=1.14\times {10}^{12} \, {s}^{-1}$ and ${\gamma }_{13}=3.14\times {10}^{12} \, {\text{s}}^{-1}$. For shake of simplicity, we have considered the carrier concentration of the system in per unit volume^43^, whose order is similar to that of 3- or 4-level quantum wells (QWs) reported earlier^53,54^. To investigate the behavior of the linear absorption of the ADQWs, we present three-dimensional (3D) profile of the imaginary part of linear or first-order susceptibility as depicted in Fig. 2a. The figure depicts the variation of $Im ({\chi }^{\left(1\right)})$ as functions of normalized probe detuning and control field Rabi frequency, ${\Delta }_{\mathrm{p}}/\updelta$ and ${\Omega }_{\mathrm{c}}/\updelta$, respectively. Here, $\updelta$ represent a normalizing constant with value $1.0\times {10}^{12} \, {\text{s}}^{-1}$. From the figure it is clear that the probe field undergoes maximum absorption at probe frequency ${\Delta }_{\mathrm{p}}/\updelta =0$ when the control field is small, i.e., at ${\Omega }_{\mathrm{c}}/\updelta \approx 0$, appearing a Gaussian type absorption peak. With increase of ${\Omega }_{\mathrm{c}}/\updelta$, the $Im ({\chi }^{\left(1\right)})$ peak divides into two absorption doublets indicating the emergence of the EIT window. Within the EIT window the absorption drastically reduces, even to zero, at higher values of ${\Omega }_{\mathrm{c}}/\updelta$, as evident from Fig. 2a. Meanwhile, to understand the behavior of linear refractive index of the medium, we present the 3D plot of ${Re( \chi }^{(1)})$ as functions of ${\Delta }_{\mathrm{p}}/\updelta$ and ${\Omega }_{\mathrm{c}}/\updelta$, as depicted in Fig. 2b. From the figure, it is seen that for smaller values of the control field, i.e. for ${\Omega }_{\mathrm{c}}/\updelta \approx 0$, the ${Re (\chi }^{(1)})$ profile possesses a dip followed by a peak with a sharp positive slope around the zero probe detuning $\left({\Delta }_{\mathrm{p}}/\updelta =0\right)$. Such a behavior of ${Re ( \chi }^{(1)})$ indicates a shift in group velocity of the probe field from normal to anomalous dispersion regime^55,56^. The behavior of ${Re (\chi }^{(1)})$ alters when ${\Omega }_{\mathrm{c}}/\updelta$ exceeds zero, i.e., for ${\Omega }_{\mathrm{c}}/\updelta >0$, the ${Re (\chi }^{(1)})$ profile follows a negative slope at around ${\Delta }_{\mathrm{p}}/\updelta =0$. This modification denotes a transition of group velocity from an anomalous to a normal dispersion. Thus, a switching from normal to anomalous dispersion regime in the system can be achieved by modulating the Rabi-frequency of the control field.

At this point, it would be worth mentioning the results of Graf et al.^57^, where the characteristics of intersubband resonances in ${\text{GaAs}}/{\text{Al}}_{0.35}{\text{Ga}}_{0.65}{\text{As}}$ multiple quantum wells (MQWs) via measuring photocurrent and photon drag were presented. They have demonstrated experimentally the effect of photon drag which led to a new method for measuring depolarization shift followed by absorption spectra. In addition, the measurement of photon drag current is performed in room temperature. On the other hand, for the present investigation the carrier concentration related to ADQWs is of the order of ${10}^{23} \, {\text{m}}^{-3}$, which is taken at temperature $77 \, {\text{K}}$^43^, Since the depolarization effect depends primarily on the electron concentration, thus the depolarization shift of the spectral position of the intersubband resonance may be expected.

**Figure 3:**
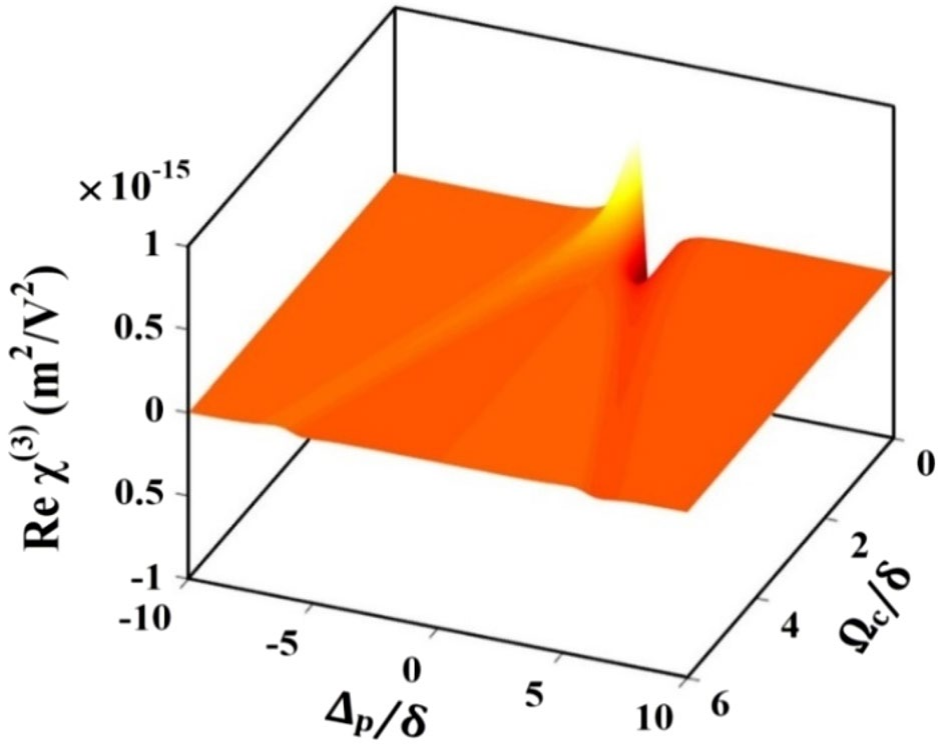
Three dimensional plot for real part of third order susceptibility $Re ({\upchi }^{\left(3\right)})$ against normalized Rabi-control frequency ${\Omega }_{c}/\delta$ and probe detuning ${\Delta }_{p}/\delta$.

We now proceed to examine the third-order susceptibility of the ADQWs leading to Kerr nonlinearity. Figure 3 depicts the 3D profile of the real part of the third-order susceptibility, $Re ({\chi }^{\left(3\right)})$ as a functions of probe detuning $\left({\Delta }_{\mathrm{p}}/\updelta \right)$ and control field $\left({\Omega }_{\mathrm{c}}/\updelta \right)$. As observed in the figure, the profile of $Re ({\chi }^{\left(3\right)})$ possesses a single peak around the ${\Delta }_{\mathrm{p}}/\updelta =0$, when ${\Omega }_{\mathrm{c}}/\delta \approx 0$. However, higher values of ${\Omega }_{\mathrm{c}}/\updelta$ (i.e. ${\Omega }_{\mathrm{c}}/\updelta >0$), adds an additional peak to $Re ({\chi }^{\left(3\right)})$ profile. The spacing between these peaks widens as the control Rabi frequency raises. Here, it is significant to note that the peak value of $Re ({\chi }^{\left(3\right)})$ is found to be of the order of ${10}^{-15}\, {\text{m}}^{2}/{\text{V}}^{2}$, which is quite larger in comparison to other nonlinear media. In addition, by accurately calibrating the control Rabi frequency, the value of $Re ({\chi }^{\left(3\right)})$ can be altered to any desired probe frequency.

### Instability gain at minimum absorption

**Figure 4:**
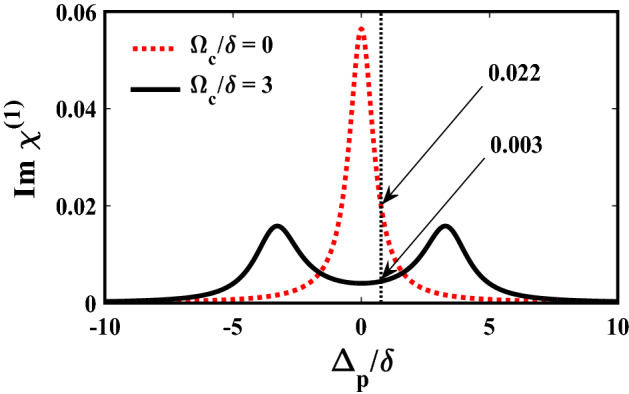
Variation of $Im ( {\upchi }^{\left(1\right)} )$ with respect to the probe detuning (normalized) for different control Rabi frequency ${\Omega }_{c}/\delta$. Vertical dashed line cuts the $Im ( {\upchi }^{\left(1\right)})$ curves at values $0.0145$ and $0.0014$, indicating that at probe detuning ${\Delta }_{\mathrm{p}}/\updelta =1.0$, absorption reduces by almost 7 times when a suitable control field is applied.

**Figure 5:**
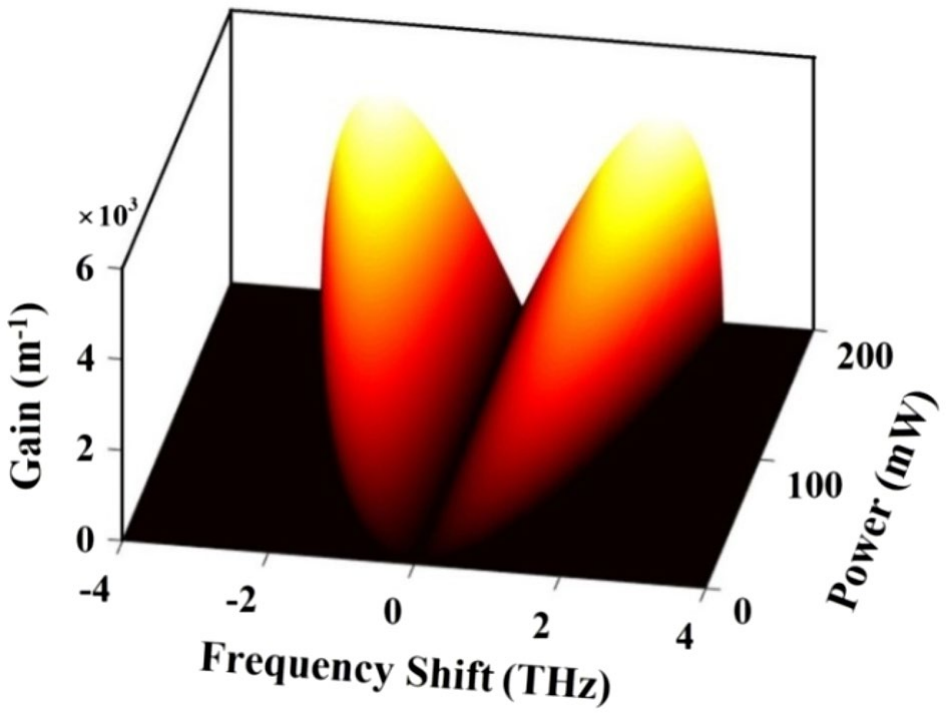
Plot of instability gain with perturbed frequency and normalized power.

Next, we proceed to investigate the gain of the modulation instability in the ADQWs, for which the same system parameters are considered as employed in sub-section (a). At this point it is important to choose an appropriate probe frequency where the dispersion and nonlinearity are high and the absorption is the least. We set the probe detuning at ${\Delta }_{p}=1.0{\times {10}^{12} \, {\text{s}}}^{-1}$, which corresponds to the wavelength $\lambda =7.28 \,  \upmu {\text{m}}$, with control field Rabi frequency ${\Omega }_{\mathrm{c}}/\updelta =3$. The absorption is found to be reduced to 0.003 from 0.022 (when no control field was applied), i.e. the probe absorption reduced almost by 7.5 times as shown in Fig. 4. At this probe detuning the nonlinear coefficient and GVD are calculated to be $W=-1.58\times {10}^{-22} \, {\text{m}}^{-1}{\text{s}}^{2}$, and ${\beta }_{2}=1.34\times {10}^{-22} \,  {\text{m}}^{-1}{\text{s}}^{2}$, respectively. Employing these parameters, we analyze the dynamics of MI gain of a continuous or quasi-continuous probe field launched into the ADQWs. We exhibit the MI gain as functions of frequency shift $(\Omega )$ and input power $\left({p}_{0}\right)$ to examine the effects of nonlinearity and dispersion on the growth of MI, as depicted in Fig. 5. The figure clearly shows that the MI growth rises linearly with power, also the frequency bandwidth is found to get widened as the power increases.

**Figure 6:**
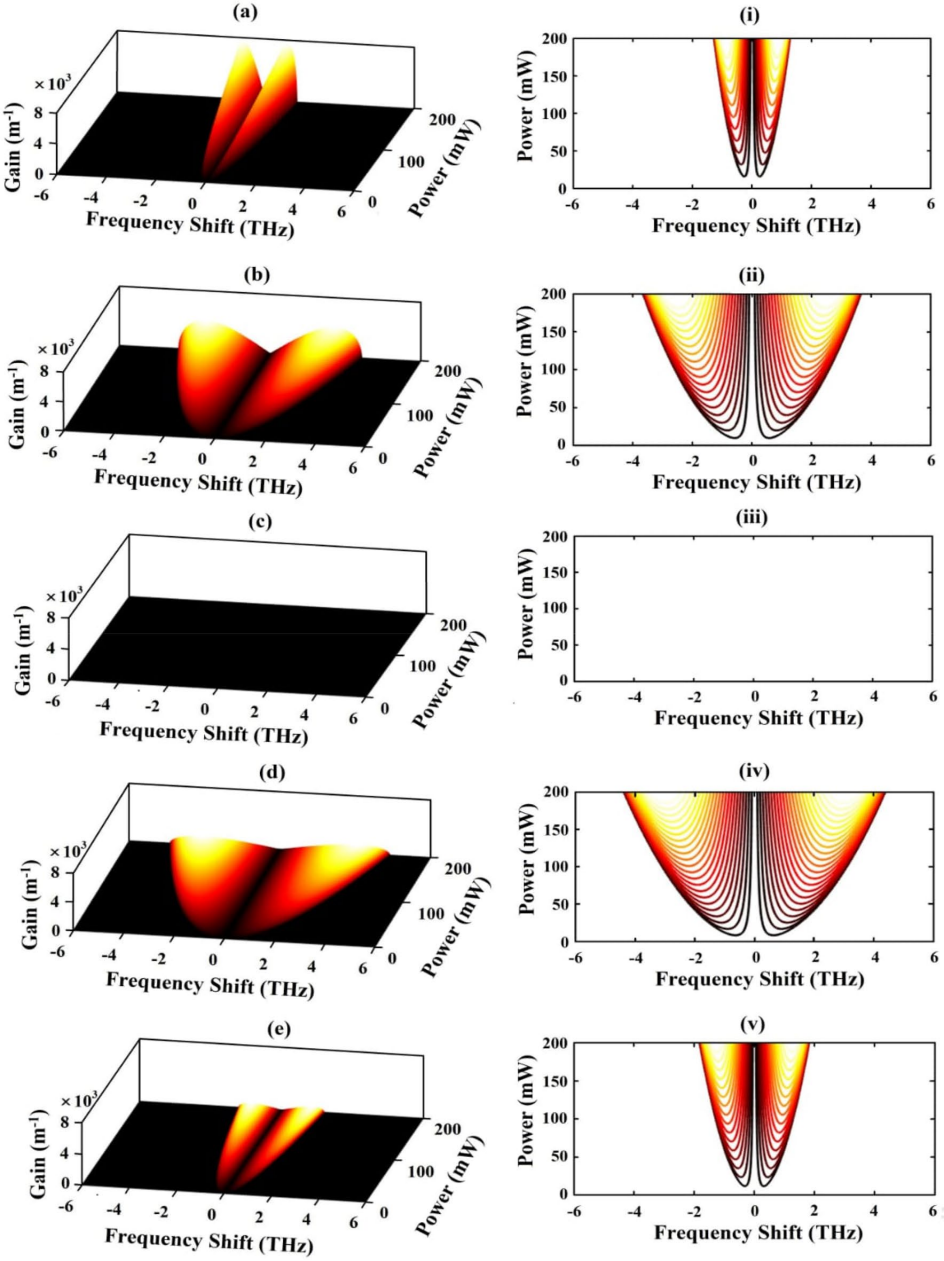
*Left panel—*Modulation instability gain spectra as factors of power and frequency shift at various control field Rabi frequencies: (**a**) ${\Omega }_{c}=0.45 \,  {\text{ps}}^{-1}$, (**b**) ${\Omega }_{c}=0.9 \,  {\text{ps}}^{-1}$, (**c**) ${\Omega }_{c}=1.5 \, {\text{ps}}^{-1}$, (**d**) ${\Omega }_{c}=2.9 \, {\text{ps}}^{-1}$, (**e**) ${\Omega }_{c}=3.5\,  {\text{ps}}^{-1}$. *Right panel—*Contour plots of MI gain spectra (**i**) to (**v**), corresponding to (**a**) to (**e**), respectively.

In the previous section, we witness that the control field Rabi frequency significantly influences the value of linear and nonlinear susceptibilities. The control Rabi frequency is expected to play a substantial impact in the development of MI gain. We therefore proceed to examine the effect of the control field Rabi frequency on the MI gain. In Fig. 6, we show the dynamics of MI gain for different values of $${\Omega }_{\mathrm{c}}/\updelta (=0.5, 0.9, 1.5, 2.9,$$ and $$3.5)$$. In this figure the left panel shows the 3D variation of MI gain as functions of power and frequency shift, while right panel shows the contour plots of the corresponding figures. Figure 6a demonstrates the linear increase in the MI gain with input power, for the case of $${\Omega }_{\mathrm{c}}/\updelta =0.45$$. Along with the gain, the bandwidth also increases linearly with power. Precisely, the bandwidth of the spectra expands to $$\pm 1.2 \, {\text{THz}}$$, at $$200 \, {\text{mW}}$$ input power. This can be observed clearly from the contour plot presented in Fig. 6i. At slightly higher value of $${\Omega }_{\mathrm{c}}/\updelta $$, for example at $${\Omega }_{\mathrm{c}}/\updelta =0.9$$, the height of the gain spectra is found to be reduced, as evident in Fig. 6b. However, the spectrum expands to quite larger values ($$\pm 3.7 \, {\text{THz}}$$, at $$200 \,  {\text{mW}}$$ input power) in comparison to the previous case, which is observed in Fig. 6ii. For minute higher values of the control Rabi frequency, say at $${\Omega }_{\mathrm{c}}/\updelta =1.5$$, no spectrum is observed throughout the range of power (as evident from Fig. 6c), which indicates that the instability gain is totally suppressed. Figure 6iii further supports the fact of suppression of MI for $${\Omega }_{\mathrm{c}}/\updelta =1.5$$. At $${\Omega }_{\mathrm{c}}/\updelta =2.9$$, the MI gain reappears with a drastic decay in the gain height, but with wide spread in the spectrum, which is evident from Fig. 6d. The spread of spectrum is recorded to extend up to $$\pm 4.2 \,  {\text{THz}}$$, at $$200 \,  {\text{mW}}$$ input power, as seen in Fig. 6iv. With further increase in $${\Omega }_{\mathrm{c}}/\updelta $$, say for $${\Omega }_{\mathrm{c}}/\updelta =3.5$$, the height of MI gain reduces to a minimum, as depicted in Fig. 6e. The spreading of the spectrum also decreases greatly (to $$\pm 1.9 \, {\text{THz}}$$, at $$200 \, {\text{mW}}$$ input power) as compared to previous case which is evident from Fig. 6v.

**Figure 7:**
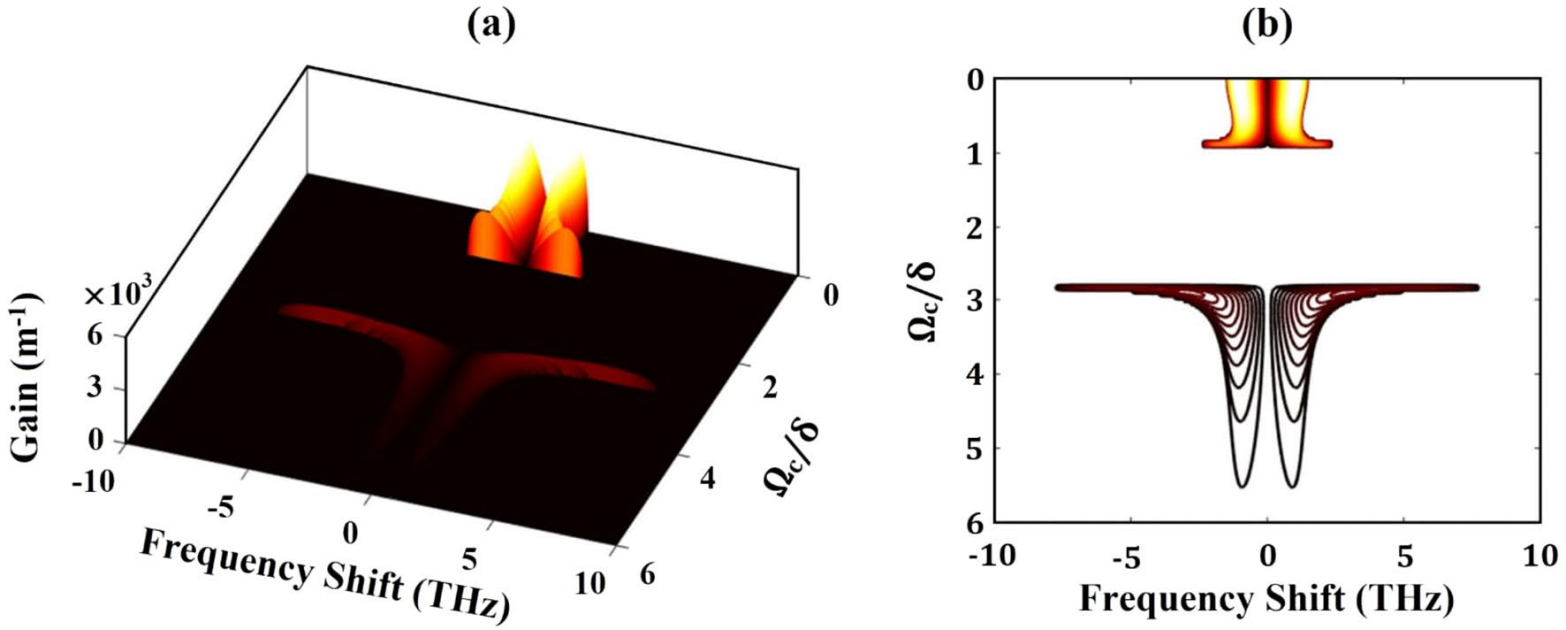
(**a**) Plot of instability gain with frequency shift and control Rabi frequency and (**b**) contour plot of the gain spectra shown in (**a**).

**Figure 8:**
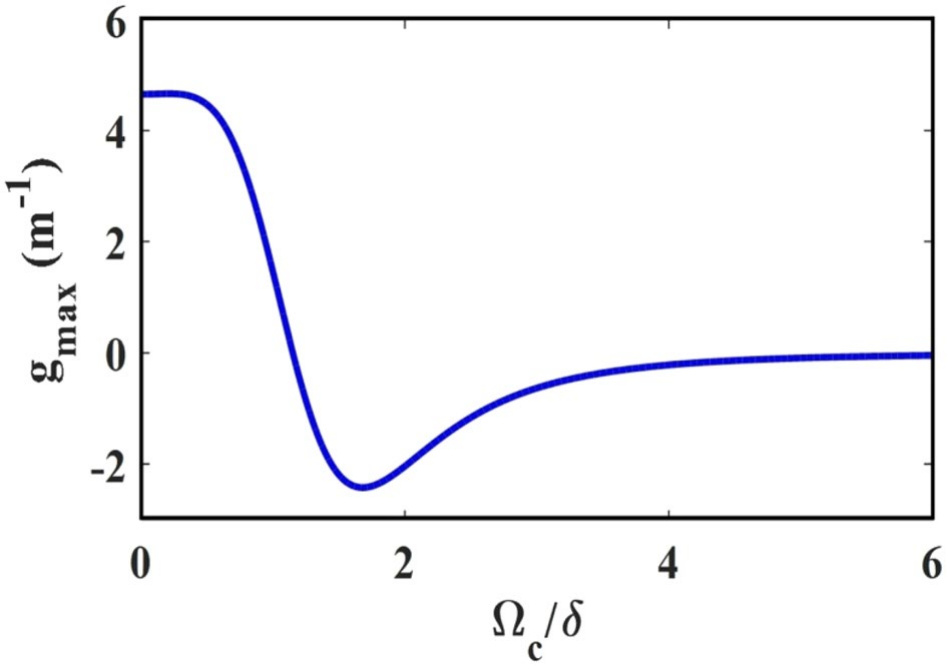
Variation of maximum gain of MI for a range of control field Rabi frequency.

The above results reveal that the height of MI gain as well as their frequency bandwidth varies randomly with the increase of control field Rabi frequency. In order to have a clear understanding of the effect of control field Rabi frequency on the stability of the MI gain, we present Fig. 7. From the figure it is clear that, a significant effect of MI can be observed for lower values of ${\Omega }_{\mathrm{c}}/\updelta$. For intermediate values of ${\Omega }_{\mathrm{c}}/\updelta$, the system is found totally stable against MI, whereas, for the higher values, the gain of MI though reappears but possesses very smaller intensities. We also plot the profile of maximum gain with respect to the control field Rabi frequency in Fig. 8. The figure amply clears that for initial values of ${\Omega }_{\mathrm{c}}/\updelta$, say for ${\Omega }_{\mathrm{c}}/\updelta \approx 0.5$ the gain is maximum. The gain falls drastically and reaches a minimum at around ${\Omega }_{\mathrm{c}}/\updelta \approx 1.6$, which then slightly get enhanced and attains a plateau of saturated value. As a result, we can assert with confidence that MI can be tuned as needed by adjusting the intensity of the control field.

In view of the light interaction with QWs, one important point to highlight is that, the adopted ADQWs for present study not only deals with the quantum confinement of electronic wave-functions in different energy subbands, but also deals with tunneling mechanism of the electronic wave-functions through the thin barriers between the wells. Hence, instead of single QWs, continuum layer in double quantum wells offers an extra advantage towards the investigation of tunneling induced quantum destructive Fano-type interference. Making use of continuum layer in ADQWs, researchers able to control the absorption of the probe and predict the versatile applications of all optical switching-based devices for optical communications purpose^43,58^. However, it is worth mentioning that, owing to the presence of continuum states in ADQWs, the gain of the MI are significantly improved as compared with single QWs and easy to control tunable characteristics of MI.

## Conclusion

In summary, we have examined the dispersive and optical nonlinear characteristics of ADQW systems in presence of EIT, where a probe pulse and a control laser beam are used in a Λ-configuration. By following density matrix approach, the expressions for linear and third-order (Kerr) susceptibilities are obtained. The presence of large Kerr susceptibility of the order of ${10}^{-15} \, {\text{m}}^{2}/{\text{V}}^{2}$ has been identified in ADQW systems. The expression for modulation instability of a continuous or quasi-continuous weak probe manifested by GVD and nonlinear coefficient is established. It is demonstrated that by modifying the value of the control Rabi-frequency allows one to control the nonlinearity of the probe field to a certain extent. Control field Rabi frequency is also seen to have influence over the EIT window as well as magnitude and behavior of linear and nonlinear susceptibility. The control field Rabi frequency could be used to limit the gain and bandwidth of the MI. An important feature of the MI is that by a suitable choice of the control field Rabi frequency, the instability could be completely suppressed. The maximum gain curve predicts that for certain values of control field Rabi frequency $({\Omega }_{c})$ the probe field is stable against modulation instability. The maximum gain curve forecasts that the probe field is stable against MI for specific values of control field Rabi frequency $({\Omega }_{c})$.

## Data Availability

All data generated or analyzed during this study are included in this published article.
